# Do Induced Responses Mediate the Ecological Interactions Between the Specialist Herbivores and Phytopathogens of an Alpine Plant?

**DOI:** 10.1371/journal.pone.0019571

**Published:** 2011-05-04

**Authors:** Gregory Röder, Martine Rahier, Russell E. Naisbit

**Affiliations:** 1 Laboratory of Evolutionary Entomology, Institute of Biology, University of Neuchâtel, Neuchâtel, Switzerland; 2 Unit of Ecology and Evolution, Department of Biology, University of Fribourg, Fribourg, Switzerland; University Copenhagen, Denmark

## Abstract

Plants are not passive victims of the myriad attackers that rely on them for nutrition. They have a suite of physical and chemical defences, and are even able to take advantage of the enemies of their enemies. These strategies are often only deployed upon attack, so may lead to indirect interactions between herbivores and phytopathogens. In this study we test for induced responses in wild populations of an alpine plant (*Adenostyles alliariae*) that possesses constitutive chemical defence (pyrrolizidine alkaloids) and specialist natural enemies (two species of leaf beetle, *Oreina elongata* and *Oreina cacaliae*, and the phytopathogenic rust *Uromyces cacaliae*). Plants were induced in the field using chemical elicitors of the jasmonic acid (JA) and salicylic acid (SA) pathways and monitored for one month under natural conditions. There was evidence for induced resistance, with lower probability and later incidence of attack by beetles in JA-induced plants and of rust infection in SA-induced plants. We also demonstrate ecological cross-effects, with reduced fungal attack following JA-induction, and a cost of SA-induction arising from increased beetle attack. As a result, there is the potential for negative indirect effects of the beetles on the rust, while in the field the positive indirect effect of the rust on the beetles appears to be over-ridden by direct effects on plant nutritional quality. Such interactions resulting from induced susceptibility and resistance must be considered if we are to exploit plant defences for crop protection using hormone elicitors or constitutive expression. More generally, the fact that induced defences are even found in species that possess constitutively-expressed chemical defence suggests that they may be ubiquitous in higher plants.

## Introduction

Plants are under constant threat of attack by herbivores and pathogens [1]. As these organisms often share the same individual plant, particularly when insects act as vectors or their feeding wounds allow establishment of pathogens, there are many opportunities for direct interactions to affect the fitness and ecology of all protagonists [2], [3]. Furthermore, plants themselves are not simply passive hosts. They participate truly in these three-way interactions, with indirect defences against herbivores, whereby damaged plants emit volatile compounds that attract the enemies of their enemies [4], and direct defences against both insects and pathogens, in which morphological structures or chemical substances are used to inhibit attack [5].

In recent decades, plant defences have grown into a vast field of investigation following the demonstration that many of these traits are only activated upon attack [6]. Two major signalling pathways are involved: infestation by biotrophic pathogens or attack by sucking insects typically activates the salicylic acid (SA) pathway and results in systemic acquired resistance (SAR) against plant diseases, while attack by herbivores or necrotrophic pathogens usually triggers the jasmonic acid (JA) pathway [7]–[10]. However, the story is more complex than this simple dichotomy, for some arthropods and pathogens induce both pathways, and cross-talk between signalling pathways is also commonly observed, typically involving reciprocal down-regulation [10]–[12]. The pathways have often been studied with a view to their exploitation in crop protection, so the majority of research has been carried out in agricultural systems [13], [14], with relatively little work on plants in their natural environment. Furthermore, few studies have been carried out on the response to induced direct defences by specialized herbivores that are able to surmount the constitutive chemical defences of their host, as in tobacco plants, where attack by the tobacco hornworm (*Manduca sexta*) induces increased endogenous JA levels, but decreased nicotine accumulation [15]. In some cases these specialists are also undeterred by induced defences [16].

As a result of these induced defences, competition mediated by changes in plant chemistry may structure diverse communities of herbivores and pathogens [3], [17]. In this study, we investigate whether induced responses of an alpine plant may mediate the interactions between its herbivores and phytopathogens. We test for induced direct defences in wild populations of the alpine plant *Adenostyles alliariae*, a species that possesses constitutive chemical defence (pyrrolizidine alkaloids) and specialist natural enemies (two species of leaf beetle, *Oreina elongata* and *Oreina cacaliae*, and the phytopathogenic rust *Uromyces cacaliae*). The host plant suffers a high proportion of leaves consumed by leaf beetles, and infection by the phytopathogenic rust in mid summer seems associated with rapid senescence of the plant. Beetle larvae grow more slowly on rust-infected plants, and both adults and larvae avoid such plants [18]. Here we test if this avoidance is a result of plant defences induced by the rust. By using reciprocal induction treatments we also investigate the potential for indirect positive or negative effects of the beetles on the rust. Artificial induction with chemical elicitors of the JA and SA pathways in natural populations was used to ask:

Do *Adenostyles alliariae* plants show induced responses?

Does artificial induction of resistance change the probability and timing of attack by *Oreina* beetles and infection by the rust?

The answers to these questions are used to examine whether the ecological interactions between *Oreina* leaf beetles and *Uromyces* rusts may be mediated by plant induced-responses.

## Materials and Methods

### Study Organisms

*Adenostyles alliariae* (Asterales: Asteraceae) is a common, perennial, subalpine and alpine plant found on damp soils near the tree-line and up to an altitude of 2800 m. Plants constitutively produce pyrrolizidine alkaloids (PAs, mainly seneciphylline and senecionine) at around 3% of dry weight [19]. These compounds are liver and lung toxins in mammalian herbivores [20], are feeding deterrents for most generalist insects [21] and also reduce attack by some fungi [22]. The herbivores *Oreina cacaliae* and *O. elongata* (Coleoptera: Chrysomelidae) are small (length 6.5 to 11.5 mm), typically metallic blue or green leaf beetles found in isolated populations throughout the Alps and Apennines, with the range of *O. cacaliae* also extending as far as the Pyrenees and Carpathians [23]–[25]. In the studied populations, *O. cacaliae* spends the entire reproductive season on *A. alliariae*, whereas *O. elongata* also feeds on *Cirsium spinosissimum* [18], [26]. The beetles are not deterred by the PAs in their host, and in fact can sequester them for their own defence [27], [28]. *Uromyces cacaliae* (Uredinales: Pucciniaceae) is a specialist microform rust of *A. alliariae*. It produces only teleutospores (teliospores), individually formed on short stems. From mid summer, the underside of infected leaves show 0.5 mm diameter brown teleutosori (telia), first covered by epidermis then free and dust-covered, forming dense groups (of 0.5 cm diameter) surrounded by a ring of yellow tissue [29], [30]. The interactions between these species are intensified by the extreme brevity of the alpine summer, with only two to three months during which they can grow and reproduce while the habitat is free of snow [31].

### Field Experiment and Treatments

Experiments were carried out at Emosson (Swiss Alps, Valais, altitude 1949 m) and La Fouly (Swiss Alps, Valais, 1587 m), inhabited by *O. elongata* and *O. cacaliae* respectively, and both showing *A. alliariae* populations infected with the rust *Uromyces cacaliae*. The two populations were studied for one season each in consecutive years. Experiments began on 27 May at La Fouly, and on 28 June at Emosson, due to the higher altitude of this site. In each population, 80 plants of *A. alliariae* were chosen at random. All were newly emerged, healthy plants with their two first leaves and no flowers. After measuring their initial heights, they were randomly assigned to one of seven treatments, mixed throughout a single patch.

(1) 20 plants were treated with acibenzolar-S-methyl (benzothiadiazole, BTH), provided as Bion solution (60 mg/l) with 50% active ingredient (Syngenta). The effect of this compound on the plant is similar to that of salicylic acid, used to induce systemic acquired resistance [13], [32]. The plants were individually sprayed four times with 0.5 ml of solution on the first day of the experiment and one week later, as suggested by the manufacturer.

(2) 20 plants were treated with methyl jasmonate, a derivative of jasmonic acid [33]. The compound is volatile, so to minimize evaporation it was mixed in pure lanolin (Riedel-de Haën) and a syringe used to produce 20 µl droplets of lanolin containing 150 µg of methyl jasmonate (Aldrich) [34]. The lowest leaf of each plant was treated, applying half of one droplet to the upper surface and half to the lower by gently spreading with a spatula. This covered an area of around 4 cm^2^ overall, representing about 2% of the leaf surface.

(3) 20 plants were treated with both compounds. The lanolin droplets were applied first, immediately followed by the Bion spray.

Control plants were split among the final four treatments: (4) five were sprayed with water, the carrier substance for BTH; (5) five were treated with one droplet of pure lanolin, the carrier for methyl jasmonate; (6) five were treated with both carriers; and finally, (7) five were left with no treatment.

Over a period of one month, the plants were monitored weekly, recording whether they showed signs of beetle attack (in the form of holes due to adult or larval feeding) and rust symptoms (easily recognizable on the upper side of leaves as a 2–3 mm diameter discoloured area with a pale yellow spot in the centre). Their height and presence of new leaves or flowers was also noted.

### Statistical Analyses

Frequencies of attack by beetles and rust at the end of the experiment were analysed in separate logistic regressions on the binomial presence/absence data. The models included terms for population (two levels), treatment (seven levels) and their interaction. It should be noted that in all analyses, the population term confounds any effects of year and beetle species, as well as plant population and geographical site. It is included in order to control for these influences while testing for treatment effects, rather than to be interpreted in itself. There were significant effects of treatment on both attack and infection rates, so the three treatments were then compared in a pairwise manner with their respective controls, by repeating the analyses with all other data excluded.

The proportions of plants remaining non-attacked at each survey were analysed using parametric survival analysis, treating the act of being attacked as “mortality”. The censorReg function was used in S-Plus 7.0 [35], coding plants that remained without attack for the full month as right-censored and all others as interval-censored (because their infestation times could only be estimated to within roughly one-week intervals). A value of 0.001 was added to data with a lower bound of zero to allow log terms to be treated. Beetle and rust attack were analysed separately in models with terms for population (Emosson and La Fouly), treatment (1 to 7), and the population by treatment interaction, entered as factors (introducing variables as strata did not significantly improve the fit). Models were compared using likelihood ratio tests. The order in which terms were added had little effect on their significance. S-Plus offers 10 possible distribution families, but all gave similar *p* values and only the analyses using a Weibull distribution are presented. This distribution was suggested by the approximately linear relationship between ln(*t*) and lnln(1/*S*(*t*)), where *S*(*t*) is the proportion of plants that remain healthy at time *t* [36].

Growth of the plants was calculated as a daily growth rate (in cm/day), by regressing height against time in days individually for each plant (these linear regressions gave a close approximation to the growth process, with *r* ^2^ values of between 0.61 and 0.99). After square root transformation, the data were analysed in an ANOVA with terms for population, treatment, and their interaction.

The numbers of leaves on plants at the end of the experiment were compared using quasi-likelihood analysis based on a Poisson distribution, which takes into account the under-dispersion of the data (mean>variance). The model included population, treatment, and interaction terms.

The probability and timing of flower production were analysed using the same methods as the beetle and rust attack, using logistic regression and survival analysis.

ANOVAs were performed using JMP 6.0 (SAS Institute, USA) while other analyses were carried out using S-Plus 7.0 [35].

## Results

### Frequency and Timing of Attack by Beetles and Rust

There were high overall rates of attack in both populations during the month of the experiment, with 74% of plants attacked by leaf beetles and 39% by the rust. Induction of the defence signalling pathways had clear effects on both natural enemies.

**Figure 1:**
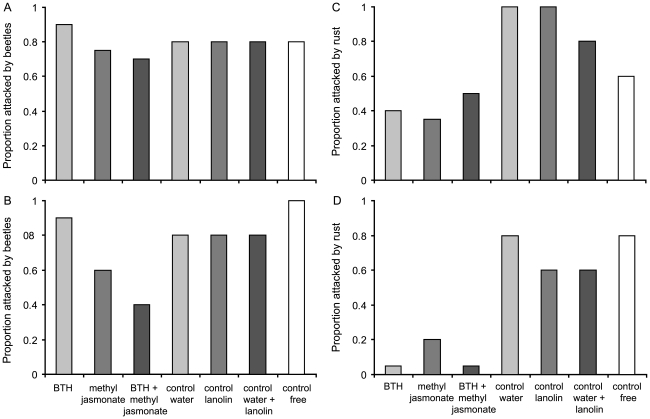
Proportions of *A. alliariae* plants attacked by *Oreina* leaf beetles and by the rust *U. cacaliae*. Graphs show data from two sites: Emosson (A and C) and La Fouly (B and D). Three groups were treated with single or combined chemical inducers of plant defences (n = 20 in each case), three others were used as their respective controls (the treatments and corresponding controls are shown in the same colour, n = 5), and finally one group was left with no manipulation (free control in white, n = 5).

The probability of attack by *Oreina* beetles was similar in the two populations but differed according to treatment (Table 1 and Figure 1). Plants treated with methyl jasmonate or with both compounds were less likely to be attacked by beetles than were their controls, while those treated with BTH suffered a higher rate of attack than the controls.

**Table 1:** Logistic regressions on the probability of attack by *Oreina* leaf beetles and infection by the rust *U. cacaliae*.

Source	DF	Deviance	Resid. DF	Resid. Dev.	P (Chi)
*leaf beetle*					
null			159	184.21	
population	1	2.076	158	182.13	0.150
treatment	6	16.404	152	165.73	0.012
pop*treatment	6	3.926	146	161.80	0.687
*rust*					
null			159	213.64	
population	1	12.960	158	200.68	<0.001
treatment	6	48.048	152	160.63	<0.001
pop*treatment	6	8.985	146	151.64	0.174

The proportion of plants infected by the rust *U. cacaliae* differed between the populations (with higher overall rates at Emosson) but there were consistent significant differences between the treatments at the two sites (Table 1 and Figure 1). Infection rates were significantly lower in all induced plants than in their respective controls.

**Figure 2:**
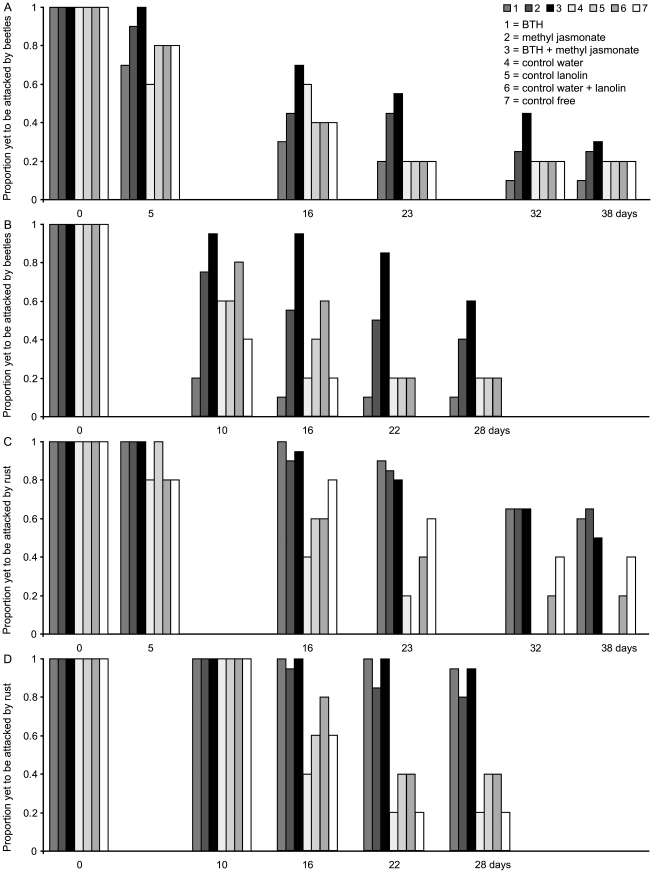
Proportions of plants still free of attack by *Oreina* leaf beetles and *Uromyces* rust over time. Graphs show data from two sites: Emosson (A and C) and La Fouly (B and D). The time axes start on the first day of experiments (day 0) and continue linearly to show the timing of the attacks. The three induced groups of plants are shown with dark colours, while their control groups are paler.

The treatments also had significant effects on the timing of beetle and rust attack in both populations (Table 2). Methyl jasmonate and doubly treated plants were attacked later by beetles, whilst the BTH treated plants were attacked more rapidly than the controls (Figure 2). For rust infection, the plants treated with the chemical inducers alone or in combination showed later signs of disease than the controls (Figure 2).

**Table 2:** Parametric survival analysis of the timing of leaf beetle and rust attack.

	Parameters	−2×LogLik	Likelihood ratio	DF	P (Chi)
*leaf beetle*					
null	2	488.77			
population	3	488.77	<0.01	1	0.985
treatment	9	456.70	32.07	6	<0.001
pop*treatment	15	450.66	6.03	6	0.419
*rust*					
null	2	384.95			
population	3	382.34	2.61	1	0.106
treatment	9	330.09	52.25	6	<0.001
pop*treatment	15	320.07	10.02	6	0.124

Beetle and rust data were analysed separately, with attack treated as “mortality”. The lines show the null model (with a single distribution location and scale parameter) and the change in log likelihood as terms for population (Emosson or La Fouly), treatment (seven levels), and the population by treatment interaction were sequentially added. The final three columns provide likelihood ratio tests of the significance of each term.

### Are Induced Plants More Successful in Growth and Reproduction?

The growth rate of plants did not differ significantly among treatments (Table S1 and Figure S1) and neither did the number of leaves at the end of the experiment (Table S2). There was also no effect of treatment on the probability of flowering during the experiment (Table S3 and Figure S2), or on the timing of flowering (Table S4 and Figure S3), although plants at Emosson were more likely to flower and did so more rapidly.

## Discussion

Our results demonstrate induced resistance in *A. alliariae*, with effects on the leaf beetles *O. elongata* and *O. cacaliae* and on the rust *U. cacaliae*. In the field, plants artificially induced with chemical signalling compounds were less likely to be attacked and were attacked later in the season. This induced resistance represents an additional defence independent of the constitutively expressed pyrrolizidine alkaloids, since the concentration of these compounds is not altered following beetle attack or rust infection [37]. Deterrence of natural populations of herbivores has only rarely been observed in previous studies, because most have used captive trials and tested for reduced herbivore performance as the measure of induction, sometimes finding no effect on specialists [16]. The effects on the timing of attack are particularly relevant in the alpine environment, allowing the plants to benefit from part of the short summer season without the challenges posed by the two antagonists.

There was also evidence for interactions between the two signalling pathways. Treatment with methyl jasmonate inhibited attack by both beetles and rust. In contrast, whilst BTH inhibited rust infection, it promoted beetle attack. This suggests that there may be asymmetric cross-talk between defences in this system and no simple mapping of jasmonic acid and salicylic acid defence pathways onto herbivore and pathogen attack, respectively [10].

The experiment revealed an ecological cost of SA induction, since it made plants more attractive to insect herbivores. Under natural conditions this cost would not be expressed, for although rust infection would normally induce the SA pathway, it also directly reduces food quality and renders plants less attractive to *Oreina* beetles [18]. This side effect of artificial induction does, however, have obvious implications for the preventive application of chemical inducers for pest control in other systems. If this were a common feature, pre-emptive induction against plant diseases would simply open the door to herbivore attack. JA induction appears to be a better candidate, since it yielded protection against both enemies. The interactions between the two signalling pathways are therefore not necessarily antagonistic as they are in many examples [9], [10], [12], but they may need to be investigated on a case by case basis before they can be exploited in practical applications. This asymmetry also raises evolutionary questions about the optimal deployment of defences and why the SA pathway is maintained, but complete answers would require a full investigation of the costs and specificity of the two pathways.

These induced responses have the potential to lead to indirect interactions between the beetles and the rust. Prior attack by beetles would be expected to induce the JA-dependent pathway and hence lead to negative effects on the rust. Since damage by beetles is not necessary for the rust to become established and nor are they implicated as vectors, the overall effect of the beetles for the rust is therefore antagonistic. In contrast, prior rust infection should induce SA-dependent pathways and increase the attractiveness of the plant to beetles. Despite this, beetles avoid infected plants in the field and larvae reared on such plants show reduced growth rates [18]. Given the results seen here, both these effects appear to be direct influences of the rust, either due to death of plant tissue or release of fungal metabolites, rather than an indirect product of the plant response. The role of induced responses in the ecological interaction is therefore asymmetric, leading to negative effects of one participant on the other, but playing no part in the reciprocal interaction.

In summary, our results show that *Adenostyles alliariae* possesses inducible resistance involving the jasmonic acid and salicylic acid pathways that is capable of reducing the rate of beetle and rust attack in the field. This provides some respite from these specialist enemies that are undeterred by the pyrrolizidine alkaloids produced by the plant. Similar tests have been made in only a limited number of systems, but given that induced defence is found even in this species that possesses constitutively expressed chemical defence, it may well be a ubiquitous feature of higher plants. Perhaps because of the short duration of the experiment, we were unable to demonstrate a concrete fitness benefit. However, the defences might be critical in repelling enemies for long enough to allow reproduction under the time stress of the alpine environment. Finally, our finding of cross effects between the pathways serves to highlight the complexity of plant responses to their enemies. More research is clearly necessary before we fully understand their role in indirect interactions between herbivores and phytopathogens and can exploit their potential for crop protection.

## Supporting Information

Figure S1 **Growth rate of plants under the seven treatments at (A) Emosson and (B) La Fouly.** Graphs show mean growth rates (in cm/day) with standard errors.
(TIF)

Figure S2 **Proportions of** ***A. alliariae*** **plants from (A) Emosson and (B) La Fouly producing flowers.** Three groups were treated with single or combined chemical inducers of plant defences, three others were used as their respective controls (the treatments and corresponding control are shown in the same colours), and finally one group was left with no manipulation (free control in white).
(TIF)

Figure S3 **Proportions of plants yet to flower over time, at (A) Emosson and (B) La Fouly.** The time axes start on the first day of experiments (day 0) and continue linearly to show the timing of flowering. The three induced groups of plants are shown with dark colours, while their control groups are paler.
(TIF)

Table S1 ANOVA on the growth rate (in cm/day) of *A. alliariae* plants in two populations under the seven treatments.
(DOC)

Table S2 Quasi-likelihood analysis based on Poisson regression of the number of leaves on plants in two populations under the seven treatments.
(DOC)

Table S3 Logistic regression on the proportion of plants flowering during the month of the experiment in the two populations and under seven treatments.
(DOC)

Table S4 Parametric survival analysis of the timing of flowering.
(DOC)
