# Supplementary figures and images for: Do Induced Responses Mediate the Ecological Interactions Between the Specialist Herbivores and Phytopathogens of an Alpine Plant?
Source: PLoS One. 2011 May 4;6(5):e19571. doi: 10.1371/journal.pone.0019571 (PMC3087800; doi:10.1371/journal.pone.0019571)

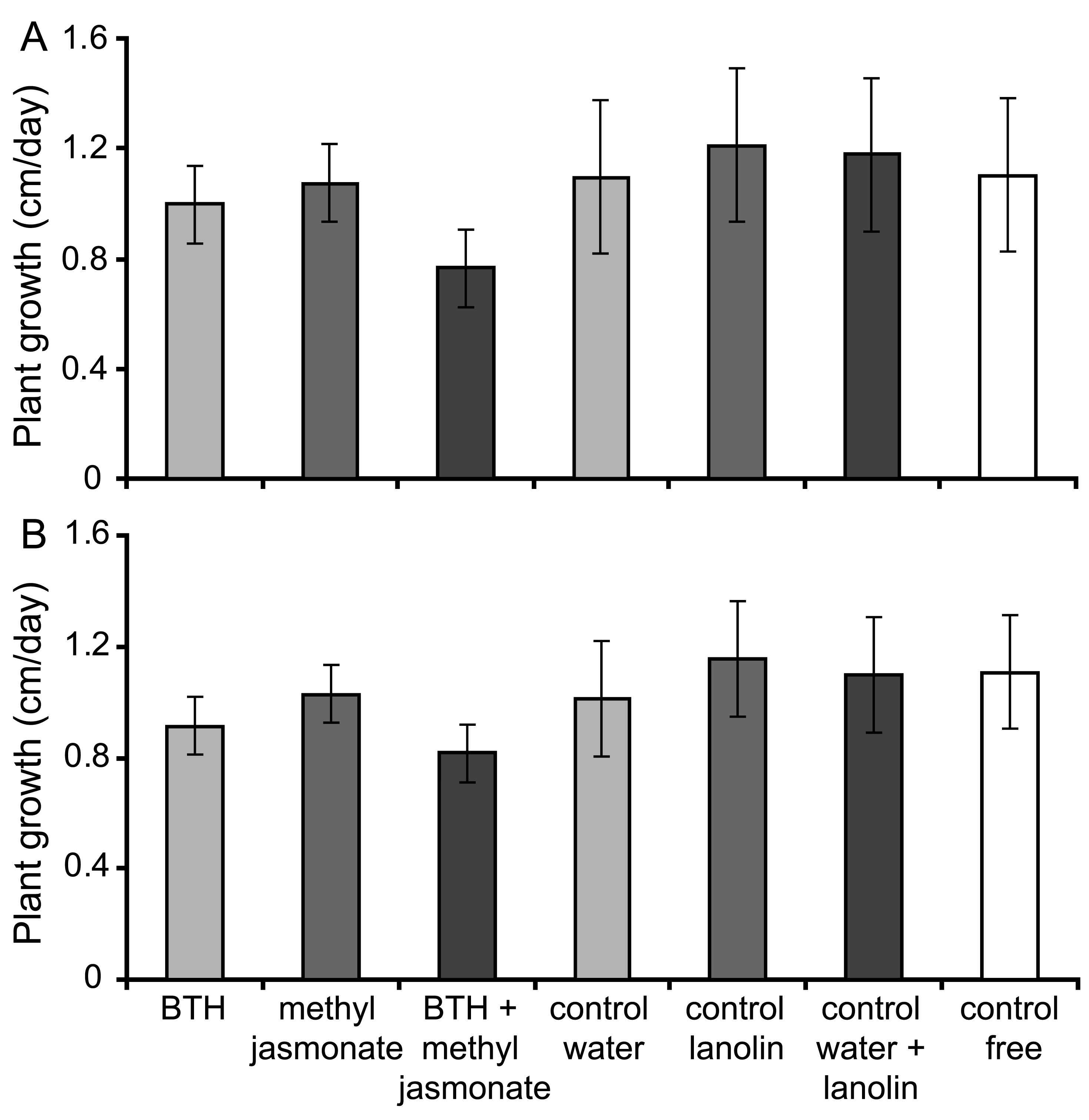

Supplement: Figure S1 — Growth rate of plants under the seven treatments at (A) Emosson and (B) La Fouly. Graphs show mean growth rates (in cm/day) with standard errors. (TIF) [file pone.0019571.s001.tif]

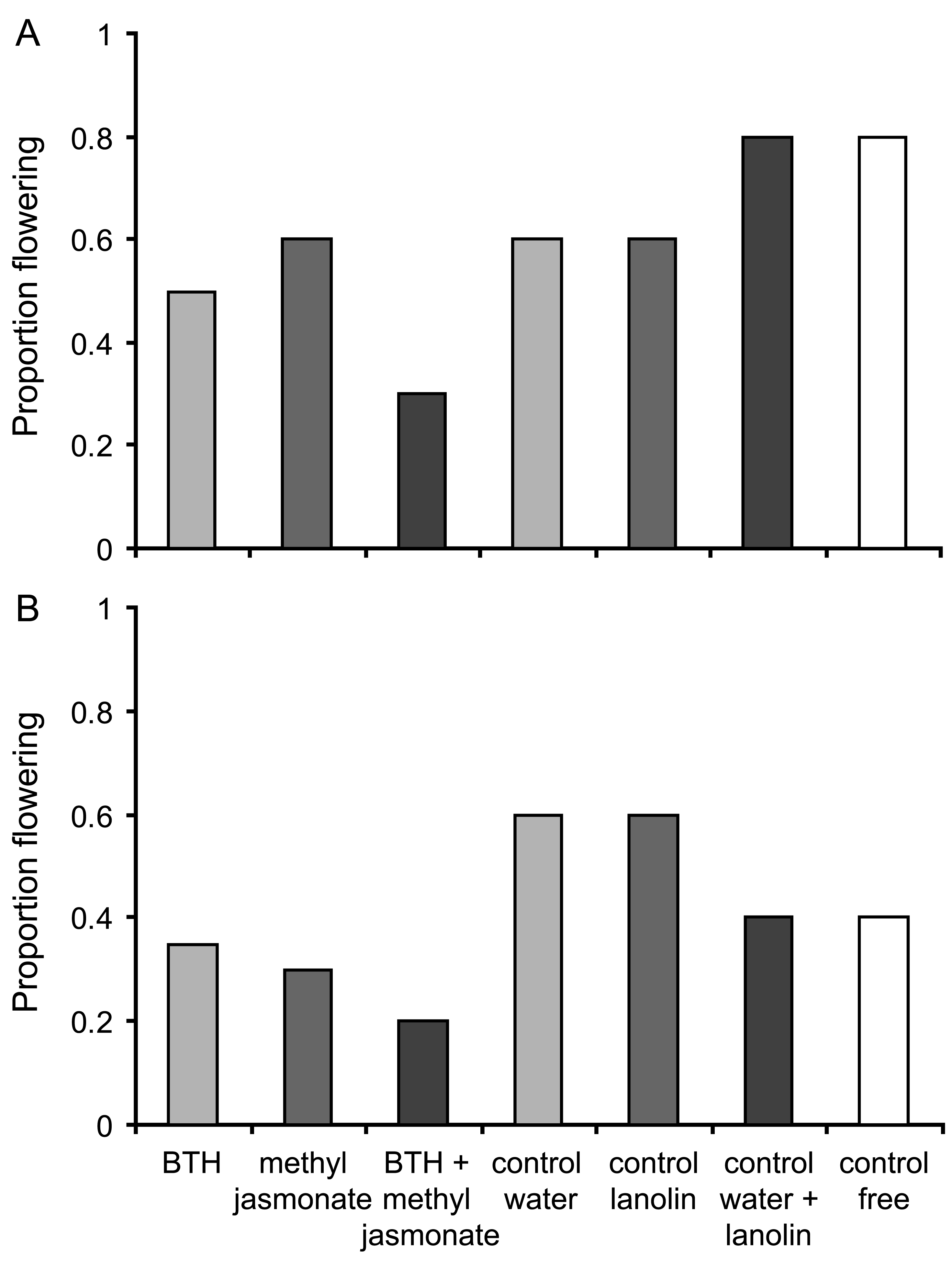

Supplement: Figure S2 — Proportions of A. alliariae plants from (A) Emosson and (B) La Fouly producing flowers. Three groups were treated with single or combined chemical inducers of plant defences, three others were used as their respective controls (the treatments and corresponding control are shown in the same colours), and finally one group was left with no manipulation (free control in white). (TIF) [file pone.0019571.s002.tif]

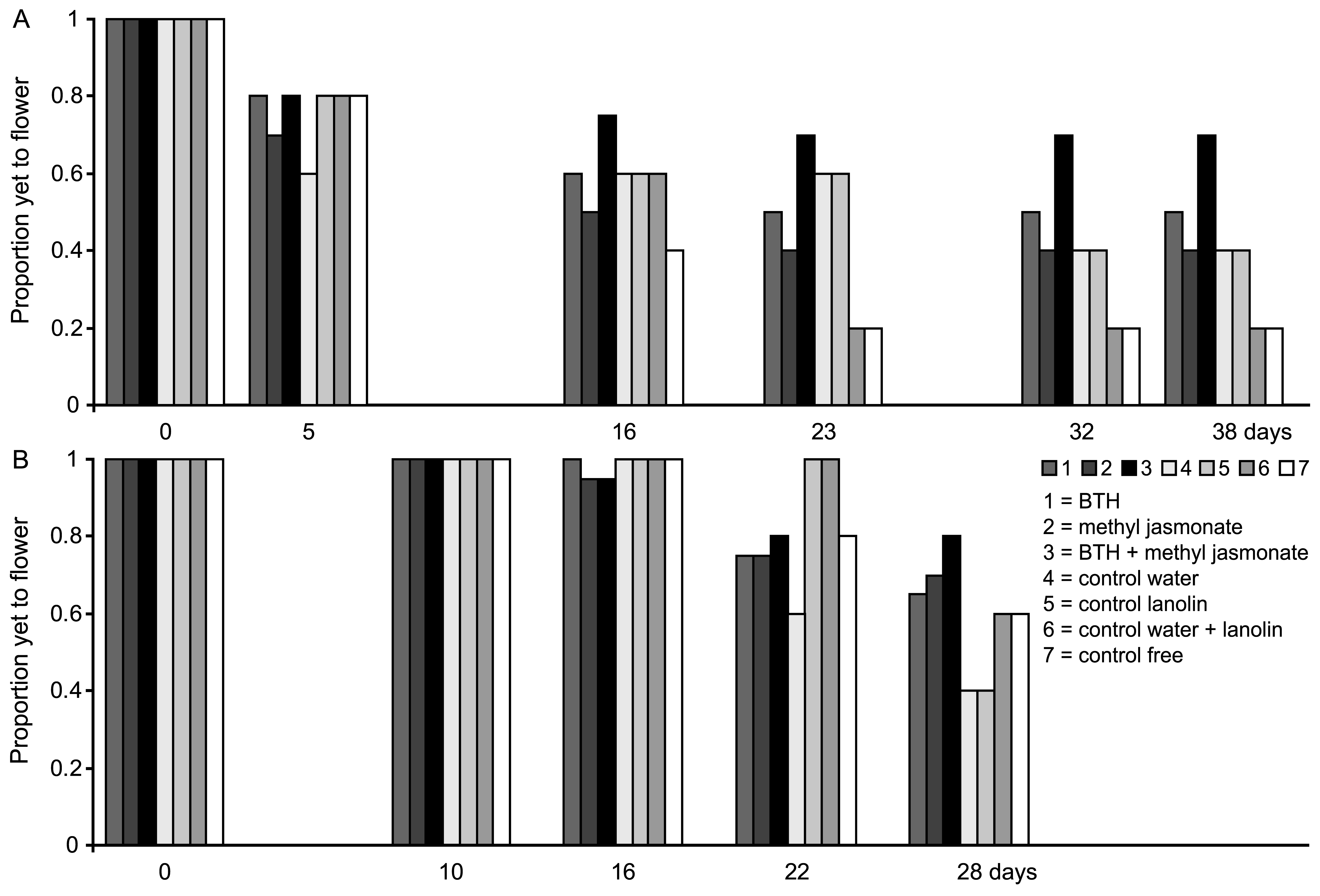

Supplement: Figure S3 — Proportions of plants yet to flower over time, at (A) Emosson and (B) La Fouly. The time axes start on the first day of experiments (day 0) and continue linearly to show the timing of flowering. The three induced groups of plants are shown with dark colours, while their control groups are paler. (TIF) [file pone.0019571.s003.tif]
