# Supplementary material for: Do Induced Responses Mediate the Ecological Interactions Between the Specialist Herbivores and Phytopathogens of an Alpine Plant?
Source: PLoS One. 2011 May 4;6(5):e19571. doi: 10.1371/journal.pone.0019571 (PMC3087800; doi:10.1371/journal.pone.0019571)
Supplement: Table S1 — ANOVA on the growth rate (in cm/day) of A. alliariae plants in two populations under the seven treatments. (DOC) [file pone.0019571.s004.doc]

**Table S1.**

ANOVA on the growth rate (in cm/day) of *A. alliariae* plants in two populations under the seven treatments.

| **Source** | **DF** | **SS** | **F** | **p-value** |
| --- | --- | --- | --- | --- |
| population | 1 | 0.003 | 0.030 | 0.863 |
| treatment | 6 | 0.581 | 1.094 | 0.369 |
| pop*treatment | 6 | 0.031 | 0.058 | 0.999 |
| error | 146 | 12.934 |  |  |
