# Supplementary material for: Do Induced Responses Mediate the Ecological Interactions Between the Specialist Herbivores and Phytopathogens of an Alpine Plant?
Source: PLoS One. 2011 May 4;6(5):e19571. doi: 10.1371/journal.pone.0019571 (PMC3087800; doi:10.1371/journal.pone.0019571)
Supplement: Table S2 — Quasi-likelihood analysis based on Poisson regression of the number of leaves on plants in two populations under the seven treatments. (DOC) [file pone.0019571.s005.doc]

**Table S2.**

Quasi-likelihood analysis based on Poisson regression of the number of leaves on plants in two populations under the seven treatments.

| **Source** | **DF** | **Deviance** | **Resid. DF** | **Resid. Dev.** | **F** | **P (F)** |
| --- | --- | --- | --- | --- | --- | --- |
| null |  |  | 159 | 26.617 |  |  |
| population | 1 | 0.003 | 158 | 26.615 | 0.014 | 0.906 |
| treatment | 6 | 1.029 | 152 | 25.586 | 0.955 | 0.458 |
| pop*treatment | 6 | 0.928 | 146 | 24.658 | 0.862 | 0.525 |
