# Supplementary material for: Do Induced Responses Mediate the Ecological Interactions Between the Specialist Herbivores and Phytopathogens of an Alpine Plant?
Source: PLoS One. 2011 May 4;6(5):e19571. doi: 10.1371/journal.pone.0019571 (PMC3087800; doi:10.1371/journal.pone.0019571)
Supplement: Table S3 — Logistic regression on the proportion of plants flowering during the month of the experiment in the two populations and under seven treatments. (DOC) [file pone.0019571.s006.doc]

**Table S3.**

Logistic regression on the proportion of plants flowering during the month of the experiment in the two populations and under seven treatments.

| **Source** | **DF** | **Deviance** | **Resid. DF** | **Resid. Dev.** | **P (Chi)** |
| --- | --- | --- | --- | --- | --- |
| null |  |  | 159 | 218.77 |  |
| population | 1 | 5.771 | 158 | 213.00 | 0.016 |
| treatment | 6 | 10.745 | 152 | 202.26 | 0.097 |
| pop*treatment | 6 | 2.438 | 146 | 199.81 | 0.875 |
