# Supplementary material for: Do Induced Responses Mediate the Ecological Interactions Between the Specialist Herbivores and Phytopathogens of an Alpine Plant?
Source: PLoS One. 2011 May 4;6(5):e19571. doi: 10.1371/journal.pone.0019571 (PMC3087800; doi:10.1371/journal.pone.0019571)
Supplement: Table S4 — Parametric survival analysis of the timing of flowering. (DOC) [file pone.0019571.s007.doc]

**Table S4.**

Parametric survival analysis of the timing of flowering.

|  | **Parameters** | **-2xLogLik** | **Likelihood ratio** | **DF** | **P (Chi)** |
| --- | --- | --- | --- | --- | --- |
| null | 2 | 432.83 |  |  |  |
| population | 3 | 427.29 | 5.54 | 1 | 0.019 |
| treatment | 9 | 417.49 | 9.80 | 6 | 0.133 |
| pop*treatment | 15 | 415.36 | 2.12 | 6 | 0.908 |

Flowering was treated as “mortality”. The lines show the null model (with a single distribution location and scale parameter) and the change in log likelihood as terms for population (Emosson or La Fouly), treatment (seven levels), and the population by treatment interaction were sequentially added. The final three columns provide likelihood ratio tests of the significance of each term.
